# Causal effects of sleep behaviors on temporomandibular disorders and pain: a bidirectional mendelian randomization study

**DOI:** 10.22514/jofph.2025.029

**Published:** 2025-06-12

**Authors:** Yuxiao Zhang, Ahsawle Ozathaley, Xiangyao Wang, Yaxin Wu, Kehan Zhang, Yuanyuan Li, Gaoshaer Nuerlan, Qilin Li, Jing Mao, Haosen Li, Shiqiang Gong

**Affiliations:** ^1^Department of Stomatology, Tongji Hospital, Tongji Medical College, Huazhong University of Science and Technology, 430030 Wuhan, Hubei, China; ^2^School of Stomatology, Tongji Medical College, Huazhong University of Science and Technology, 430030 Wuhan, Hubei, China; ^3^Hubei Province Key Laboratory of Oral and Maxillofacial Development and Regeneration, 430030 Wuhan, Hubei, China

**Keywords:** GWAS, Temporomandibular disorder, Sleep behaviors, Mendelian randomization

## Abstract

**Background**: Temporomandibular disorder (TMD) is a prevalent condition associated with pain and dysfunction of the temporomandibular joint (TMJ) and surrounding structures. Sleep disturbances are common in TMD patients, yet the bidirectional causal relationship between specific sleep behaviors and TMD remains unclear. This study aimed to investigate this relationship using a two-sample Mendelian randomization (MR) approach. **Methods**: A bidirectional two-sample MR analysis was conducted using genome-wide association study (GWAS) summary statistics for seven sleep behaviors and TMD/TMD related pain (TMD-pain). Single nucleotide polymorphisms (SNPs) were used as instrumental variables. The analysis included data from the UK Biobank and FinnGen Consortium, focusing on individuals of European ancestry. Statistical methods included inverse variance weighting (IVW), MR-Egger regression and sensitivity analyses to ensure robust findings. **Results**: The analysis identified that genetically predicted “morning person” status reduced the risk of TMD (β: −0.173, *p* = 0.014), while longer sleep duration decreased the risk of TMD-pain (β: −0.382, *p* = 0.015). In reverse MR analysis, TMD-pain increased the risk of insomnia, obstructive sleep apnea (OSA) and sleep disorders (*p* < 0.05). No significant associations were found between other sleep behaviors and TMD or TMD-pain. **Conclusions**: This study demonstrates a bidirectional relationship between sleep behaviors and TMD. Being a morning person and having longer sleep duration protect against TMD and TMD-pain, respectively, while TMD-pain increases the risk of certain sleep disorders. These findings highlight the importance of addressing sleep issues in TMD management.

## 1. Introduction

Temporomandibular disorder (TMD) is a chronic condition characterized by pain and/or dysfunction in the temporomandibular joint (TMJ) and its associated structures, including the masticatory muscles. TMD related pain (TMD-pain) ranks as the second most prevalent cause of orofacial discomfort worldwide [1]. Although TMD often presents as a self-limited condition with mild to moderate pain, it frequently coexists with comorbidities such as headaches, otologic symptoms, chronic fatigue and sleep disturbances. Poor sleep quality, highly prevalent in TMD patients, significantly impacts the disorder’s prognosis [2]. Sleep is vital for maintaining physiological functions and memory consolidation, yet sleep disorders, including fragmentation, hypersomnia and circadian rhythm disturbances, are increasingly common worldwide, affecting approximately 27% of the global population [3]. Epidemiological studies show that up to 15% of adults suffer from TMD, with a retrospective study of 1488 patients with painful TMD (63.8% female), using chart review and self-reported questionnaires, finding that 78.4% of patients reported poor sleep quality, highlighting the significant prevalence of sleep disturbances in this population [4]. Notably, insomnia and sleep apnea are the most prevalent sleep disturbances in this population, and inadequate sleep quality is associated with a 32% increased risk of developing TMD de novo, along with higher levels of pain [5, 6]. Given the lack of a definitive cure for TMD, understanding the role of modifiable risk factors, such as sleep disturbances, is essential for early intervention and improving treatment strategies.

The relationship between TMD and sleep quality is well-established. A clinical cohort study by Lee *et al*. [7] showed that individuals with chronic TMD are more likely to develop obstructive sleep apnea (OSA) and other sleep disorders. Notably, the association between TMD and sleep is particularly strong for the painful form of TMD, which correlates with reduced nocturnal sleep duration [8]. Research during the COVID-19 pandemic further revealed differential impacts of sleep disturbances on TMD symptoms: painful TMD patients experienced increased sleep latency and greater dependence on sleep medication, while painless TMD patients had worsened jaw dysfunction, though the incidence of TMD itself remained unchanged [9]. Additionally, a systematic review of sleep disturbances in temporomandibular joint osteoarthritis (TMJ-OA) patients found that these individuals often report poor sleep quality, but the evidence remains insufficient to establish a clear causal relationship [10]. These findings underscore the need for further research to explore the mechanisms that mediate the complex interaction between sleep and TMD.

Mendelian randomization (MR) analysis is an advanced methodological framework that leverages single nucleotide polymorphisms (SNPs) as genetic instrumental variables to serve as proxies for exposure factors, exploiting the random allocation of alleles during meiosis [11]. This approach inherently mitigates the impact of reverse causation and confounding variables, thereby providing a more robust basis for causal inferences than traditional observational studies [11, 12]. However, despite its strengths, MR is not without limitations. Potential pleiotropic effects where genetic variants influence multiple traits independently of the hypothesized causal pathway could introduce bias and affect the validity of results. To address this, we employ multiple sensitivity analyses to detect and mitigate pleiotropy, ensuring the robustness of our findings.

In the present study, we employed a two-sample bidirectional MR analysis to explore the potential causal relationship between seven distinct sleep behaviors and both TMD and TMD-pain, while also examining the directionality of these associations. Initially, we extracted validated genetic variants associated with these sleep behaviors from a comprehensive database of publicly available genome-wide association studies (GWAS). We then assessed their relationship with TMD and TMD-pain. By reversing the roles of exposure and outcome, our study aims to provide deeper insights into the complex association between sleep behaviors and TMD, emphasizing the crucial role that sleep patterns and schedules may play in the onset and progression of TMD.

## 2. Methods

### 2.1 Study design

We conducted a two-sample Mendelian Randomization (MR) study, using SNPs as genetic proxies to assess causality between self-reported sleep phenotypes and TMD, including TMD-pain. Given that SNPs are randomly allocated at conception, they are not subject to confounding by environmental factors, thus offering a robust framework for causal inference [13]. The overall study design and methodology are depicted in Fig. 1. The increasing availability of open-access genetic databases has significantly enhanced the feasibility of MR studies. All summary-level GWAS data utilized in this research are publicly accessible.

**Fig. 1.** Schematic diagram of causal inferences between sleep behaviors and TMD/TMD-pain. LD: linkage disequilibrium; BWMR: Bayesian Weighted Mendelian Randomization; TMD-pain: TMD related pain; SNP: single nucleotide polymorphism.

### 2.2 Data sources

The summary-level GWAS data employed in this study were sourced from the FinnGen Consortium and the UK Biobank. To enhance the validity and minimize genetic population structure confounding, we restricted our analysis to individuals of European ancestry. Table 1 (Ref. [14, 15, 16, 17, 18]) summarizes the data sources, sample sizes, and the definitions of the relevant phenotypes.

**Table 1.** Detailed information on the GWAS datasets used in this study.

Trait	Phenotype definition	Consortium	Sample size	Population
Daytime napping	Participants were asked, “Do you take naps during the day?” Responses included “never/rarely”, “sometimes” and “usually”, which were coded as 1, 2 and 3, respectively, to reflect increasing frequency of napping [15].	UK Biobank	452,633 individuals	European
Daytime sleepiness	Participants were asked, “How likely are you to doze off or fall asleep during the day when you don’t intend to, such as while working, reading or driving?” Response options included “never”, “sometimes”, “often” and “always”, which were encoded from 1 to 4 to reflect the increasing severity of daytime sleepiness [14].	UK Biobank	452,071 individuals	European
Insomnia	To evaluate the frequency of insomnia symptoms, participants were asked, “Do you have trouble falling asleep at night or waking up in the middle of the night?” The response options were “never/rarely”, “sometimes” and “usually”. We then created a binary variable for insomnia frequency, coding “usually” as 1 and combining “never/rarely” and “sometimes” as 0 [17].	UK Biobank	Cases: 66,976; Controls: 141,982	European
Morning person (binary chronotype)	A person’s chronotype, which indicates their preference for sleeping earlier or later, defines their circadian rhythm. Genetic association estimates for chronotype were derived from a genome-wide association study (GWAS) involving 449,734 individuals of European ancestry [18].	UK Biobank	449,734 individuals	European
OSA	Cases: FinnGen code G6_SLEEPAPNO Controls: not classified as case	FinnGen release 9	Cases: 38,998; Controls: 336,659	European
Sleep disorder	Cases: FinnGen code F5_SLEEP_NOS Controls: not classified as case	FinnGen release 9	Cases: 44,299; Controls: 329,251	European
Sleep duration	Participants were asked, “How many hours of sleep do you get in a 24-hour period?” Responses were recorded in whole hours, allowing only integer values [16].	UK Biobank	446,118 individuals	European
TMD	Cases: FinnGen code TEMPOROMANDIB Controls: not classified as case	FinnGen release 9	Cases: 5668; Controls: 205,355	European
TMD-pain	Cases: FinnGen code DENTAL_TMD Controls: not classified as case	FinnGen release 9	Cases: 10,303; Controls: 366,974	European

*OSA: obstructive sleep apnea; TMD: Temporomandibular disorder; TMD-pain: TMD related pain.*

Genetic associations for seven sleep-related traits were extracted from GWAS datasets derived from the UK Biobank and the FinnGen research project: daytime sleepiness [14] (n = 452,071), daytime napping [15] (n = 452,633), sleep duration [16] (n = 446,118), insomnia [17] (n = 208,958, comprising 66,976 cases and 141,982 controls), morning person [18] (n = 449,734), OSA (n = 375,657, including 38,998 cases and 336,659 controls) and sleep disorder (n = 373,550, including 44,299 cases and 329,251 controls).

The summary statistics for TMD and TMD-pain were obtained from the ninth data release of the FinnGen Consortium. This dataset includes 5668 cases of TMD and 205,355 controls, along with 10,303 TMD-pain cases and 366,974 controls. To clarify, the classification of these groups adheres to internationally recognized diagnostic criteria, specifically the International Classification of Diseases, 10th Revision (ICD-10) codes M79.1, K07.60 and K07.63 [19]. The TMD group in our study encompasses all forms of temporomandibular disorders, including both pain-related and non-pain-related conditions. In contrast, the TMD-pain group is specifically restricted to individuals with pain-associated TMD, including myalgia. This distinction ensures that our analysis effectively captures differences between general TMD cases and those where pain is a defining feature.

### 2.3 Selection of the genetic instrumental variables

We selected instrumental variables (IVs) associated with each exposure using a genome-wide significance threshold of *p* < 5 × 10^−8^. For OSA, sleep disorder, TMD and TMD-pain, we relaxed this threshold to *p* < 5 × 10^−6^ due to an insufficient number of SNPs available at the more stringent level. This adjustment was necessary to ensure sufficient statistical power, as a lower threshold is commonly used in studies with limited SNP availability [20, 21, 22]. Independent SNPs were identified using the PLINK clumping method in the TwoSampleMR package, applying a linkage disequilibrium (LD) threshold of *r*^2^ < 0.001 and a window size of 10,000 kb. The reference panel for LD was the 1000 Genomes European dataset. SNP effect estimates for each instrument were aligned with those for the corresponding outcomes. Palindromic SNPs, which introduce ambiguity, were excluded from the analysis.

To assess the strength of the selected instrumental variables, we computed the *F*-statistic for each SNP associated with the exposure, using the formula:

$$F=\frac{r^{2}(N-k-1)}{k(1-r^{2})}$$

Where N is the sample size, k is the number of instrumental variables, and *r*^2^ is the proportion of variance explained by the SNP. SNPs with an *F*-statistic < 10 were considered weak instruments and were excluded from further analysis. This threshold was chosen based on recommendations from previous studies, aiming to reduce the impact of weak instrument bias on causal effect estimates [23, 24, 25]. This process ensured a robust set of instrumental variables for subsequent MR analysis. Detailed information regarding the selected instrumental variables is provided in **Supplementary Table 1**.

### 2.4 Data analysis

Statistical analyses were conducted using RStudio version 4.2.2 (Posit, Boston, MA, USA). The primary tool for MR analysis was the “TwoSampleMR” R package (version 0.5.6), which integrates five advanced MR methods, with inverse variance weighting (IVW) being particularly pivotal. IVW excludes the intercept in regression models, employs the inverse of the squared standard error as a weighting factor, and assumes that all SNPs used as instruments are both independent and valid. This approach serves as the primary method for estimating the association between genetically predicted sleep behaviors and TMD, as well as its related pain.

To address potential polygenic structure and pleiotropy, we additionally utilized the Bayesian Weighted Mendelian Randomization (BWMR) method to further substantiate the causal relationships. Given the multiple comparisons in our analysis, we applied the false discovery rate (FDR) correction using the Benjamini-Hochberg method to adjust for multiple testing. Associations with an adjusted *p*-value (*q*-value) < 0.05 were considered statistically significant to minimize the risk of false positives. The confirmation of associations between sleep behaviors and TMD/TMD-pain was based on two stringent criteria: (1) the 95% confidence interval (CI) from IVW estimates did not include 1 or 0, and the FDR-adjusted *q*-value was < 0.05; (2) consistency in the direction of the effect across the six MR methods employed.

To evaluate SNP heterogeneity, Cochran’s *Q* test was performed. A *p*-value < 0.05 was considered indicative of significant heterogeneity, necessitating the exclusion of SNPs with the smallest *p*-values or the adoption of a random-effects model to assess the MR effect. Furthermore, the intercept of MR-Egger regression was calculated to detect potential horizontal pleiotropy in the instrumental variables [26]. The absence of pleiotropy was confirmed if the intercept *p*-value exceeded 0.05.

To ensure the robustness of the IVW-based results, we employed the “MR-PRESSO (Mendelian Randomization Pleiotropy RESidual Sum and Outlier)” R package to identify and exclude outliers [26]. The stability of the results was further confirmed using the leave-one-out sensitivity analysis, ensuring the reliability of our findings.

## 3. Results

### 3.1 Causal effects of sleep behaviors on TMD and TMD-pain

We analyzed the association between seven sleep characteristics (designated as exposure variables) and TMD and TMD-pain (designated as outcome variables). The results of this analysis are presented in Fig. 2A–C and **Supplementary Table 2**. The analysis revealed that individuals with a genetic predisposition toward morningness had a 15.9% lower relative risk of developing TMD (β = −0.173, 95% CI: −0.311 to −0.034, *p* = 0.014, *q* = 0.031, Odds Ratio (OR) = 0.841). The 95% confidence interval (CI) is entirely below 0, indicating that morningness has a statistically significant protective effect against the development of TMD. This finding suggests that maintaining circadian rhythm stability may confer a protective effect against TMD. Additionally, genetically predicted longer sleep duration was significantly associated with a lower risk of TMD-pain (β = −0.382, 95% CI: −0.689 to −0.075, *p* = 0.015, *q* = 0.035, OR = 0.682), corresponding to an approximately 31.8% reduction in relative risk. The 95% CI is entirely below 0, suggesting that longer sleep duration has a significant protective effect against TMD-pain. This finding indicates that insufficient sleep can heighten pain sensitivity and promote chronic inflammation, thereby contributing to the pathogenesis and exacerbation of TMD-pain.

**Fig. 2.** Causal effects of sleep behaviors on TMD and TMD-pain. (A) Heatmap illustrating the causal effects of genetically predicted sleep behaviors on TMD across different MR methods. (B) Heatmap illustrating the causal effect of sleep characteristics on TMD-pain. Heatmap illustrating the causal effects of genetically predicted sleep behaviors on TMD-pain across different MR methods. Sleep behaviors that showed significant associations with TMD or TMD-pain (IVW *p* < 0.05) are labeled in red. (C) Forest plot of MR estimates (IVW *p* < 0.05) for the causal effect of sleep behaviors on TMD and TMD-pain. MR: Mendelian randomization; BWMR: Bayesian Weighted Mendelian Randomization; OSA: obstructive sleep apnea; TMD: Temporomandibular disorder; TMD-pain: TMD related pain; CI: confidence interval.

Subsequent analyses using the MR-Egger method indicated that these associations were not confounded by significant pleiotropic effects (morning person: *p* = 0.898; sleep duration: *p* = 0.857), and no evidence of heterogeneity was found for either characteristic (morning person: *p* = 0.163; sleep duration: *p* = 0.105), as shown in Table 2 and **Supplementary Table 3**. The MR-PRESSO analysis showed no outliers among the SNPs associated with morning person and sleep duration, emphasizing the robustness of our findings. The leave-one-out analysis further confirmed the stability of our results, as illustrated in **Supplementary Figs. 1,2**. Consistent trajectories of β values were observed across multiple analysis methods, including MR-Egger analysis, simple model analysis, weighted median analysis and weighted model analysis. The scatter plot in Fig. 3A illustrates the causal relationship between being a morning person and TMD outcomes, reinforcing our confidence in the inverse relationship between morning person and TMD incidence. Similarly, the scatter plot in Fig. 3B illustrates the causal relationship between sleep duration and TMD-pain outcomes, strengthening our confidence in the inverse relationship between sleep duration and TMD-pain incidence. Additionally, our analysis did not find any significant associations between other sleep characteristics and the incidence of TMD or TMD-pain.

**Table 2.** Heterogeneity and pleiotropy in MR estimates (IVW *p* < 0.05) between sleep behaviors and TMD/TMD-pain.

Exposure	Outcome	Heterogeneity (Cochrane’s *Q*)		Pleiotropy		
		IVW_*p*val	MR-Egger_*p*val	MR-Egger_intercept	MR-Egger_*p*val	MR-PRESSO Global Test *p*val
Morning person	TMD	0.153	0.163	−0.001	0.898	0.144
Sleep duration	TMD-pain	0.092	0.105	0.002	0.857	0.152
TMD-pain	Insomnia	0.374	0.420	0.013	0.945	0.435
TMD-pain	OSA	0.270	0.240	0.006	0.325	0.103
TMD-pain	Sleep disorder	0.137	0.134	0.005	0.291	0.063

*TMD: Temporomandibular disorder; OSA: obstructive sleep apnea; IVW: inverse variance weighting; MR: Mendelian randomization; TMD-pain: TMD related pain; MR-PRESSO: Mendelian Randomization Pleiotropy RESidual Sum and Outlier; pval: p value.*

**Fig. 3.** Scatterplot of the causal effect of sleep behaviors on TMD and TMD-pain (IVW *p* < 0.05). (A) Scatterplot of the causal effect of morning person on TMD. (B) Scatterplot of the causal effect of sleep disorder on TMD-pain. Each point represents a single nucleotide polymorphism (SNP) used as an instrumental variable in the MR analysis, with horizontal and vertical error bars indicating standard errors for the SNP’s effect on the exposure and outcome, respectively. MR: Mendelian randomization; SNP: Single nucleotide polymorphism; TMD: Temporomandibular disorder; TMD-pain: TMD related pain.

### 3.2 Causal effects of TMD and TMD-pain on sleep behaviors

In the reverse MR analysis, TMD and TMD-pain were set as exposure variables, while seven sleep behaviors were the outcome variables. The main results are presented in Fig. 4A–C and **Supplementary Table 2**, indicate that genetically predicted TMD-pain was significantly associated with an increased risk of insomnia (β = 0.140, *p* = 0.013, *q* = 0.027, OR = 1.150), OSA (β = 0.063, *p* = 0.013, *q* = 0.029, OR = 1.065) and sleep disorders (β = 0.070, *p* = 0.001, *q* = 0.010, OR = 1.073). Specifically, TMD-pain was found to increase the relative risk of insomnia by approximately 15.0%, OSA by 6.5% and sleep disorders by 7.3%. The 95% CI for all these associations is entirely above 0, providing strong evidence that TMD-pain significantly increases the risk of insomnia, OSA and sleep disorders.

To validate these associations, we performed additional sensitivity analyses. MR-Egger analyses did not suggest potential horizontal pleiotropy (insomnia: *p* = 0.945; OSA: *p* = 0.325; sleep disorder: *p* = 0.291) or heterogeneity among SNPs for the exposure factors (insomnia: *p* = 0.420; OSA: *p* = 0.240; sleep disorder: *p* = 0.134). Additionally, MR-PRESSO analysis did not identify any outliers among the SNPs related to the exposure factors, as detailed in Table 2 and **Supplementary Table 3**. The leave-one-out analysis confirmed the robustness of our findings, as illustrated in **Supplementary Figs. 3,4**. The β values from the MR-Egger, simple model, weighted median and weighted model analyses were directionally consistent, further supporting the robustness of the results. Fig. 5 displays scatter plots that elucidate the causal relationship between TMD-pain and the sleep characteristics of insomnia, OSA and sleep disorder. It is also noteworthy that our study found no significant correlation between genetic susceptibility to TMD and the seven sleep behaviors, nor any evidence that TMD-pain was associated with the other four sleep behaviors. This provides valuable insights and references regarding the impact of TMD occurrence and its accompanying pain on the sleep behaviors of patients.

**Fig. 4.** Causal effects of TMD and TMD-pain on sleep behaviors. (A) Heatmap illustrating the causal effect of TMD on sleep characteristics. (B) Heatmap illustrating the causal effect of TMD-pain on sleep characteristics. Heatmap illustrating the causal effects of genetically predicted sleep behaviors on TMD-pain across different MR methods. Sleep behaviors that showed significant associations with TMD or TMD-pain (IVW *p* < 0.05) are labeled in red. (C) Forest plot of MR estimates (IVW *p* < 0.05) for the causal effect of TMD and TMD-pain on sleep characteristics. MR: Mendelian randomization; BWMR: Bayesian Weighted Mendelian Randomization; OSA: obstructive sleep apnea; TMD: Temporomandibular disorder; TMD-pain: TMD related pain.

**Fig. 5.** Scatterplot of the causal effect of TMD and TMD-pain on sleep behaviors (IVW *p* < 0.05). (A) Scatterplot of the causal effect of TMD-pain on insomnia. (B) Scatterplot of the causal effect of TMD-pain on OSA. (C) Scatterplot of the causal effect of TMD-pain on sleep disorder. Each point represents a single nucleotide polymorphism (SNP) used as an instrumental variable in the MR analysis, with horizontal and vertical error bars indicating standard errors for the SNP’s effect on the exposure and outcome, respectively. MR: Mendelian randomization; SNP: Single nucleotide polymorphism; TMD: Temporomandibular disorder; TMD-pain: TMD related pain.

## 4. Discussion

In this bidirectional two-sample Mendelian randomization study, we systematically evaluated the associations between seven genetically predicted sleep behaviors (insomnia, sleep duration, daytime sleepiness, daytime napping, morning person, OSA and sleep disorder) and TMD/TMD-pain. Our findings suggest that a genetic predisposition to being a morning person reduces the risk of TMD. Additionally, an inverse causal relationship exists between genetically predicted sleep duration and TMD-pain. Conversely, reverse MR results indicate that TMD-pain increases the risk of insomnia, OSA and sleep disorders. No significant associations were found between other sleep behaviors and TMD/TMD-pain.

The human body operates under the influence of an internal “biological clock”, which consists of a network of genes and the proteins they produce. This clock governs various physiological processes throughout the body. A person’s chronotype, which reflects this biological timing system, dictates their natural inclination toward activity, wakefulness or sleep at certain times within a 24-hour period. Chronotypes are generally categorized into three types: morningness (better suited for daytime activities), eveningness (better suited for nighttime activities) and intermediate profile (adaptable to both day and night tasks) [27]. The observed protective effect of morningness against TMD may stem from circadian alignment with environmental light-dark cycles, optimizing the expression of clock genes (*e.g.*, *CLOCK*, *PER1/2*) that regulate inflammatory pathways [28]. Morning chronotypes are associated with more stable cortisol rhythms and higher melatonin amplitude, which may mitigate neuroinflammation and nociceptive signaling in the trigeminal system—key contributors to TMD pathogenesis [29]. Research has increasingly linked chronotypes with various health issues, including disorders of the sleep-wake rhythm, hypertension, diabetes and obesity [30]. The study of chronotypes in relation to oral health, however, has been limited, with most research focusing on conditions like bruxism. Recent findings indicate a significant association between chronotypes and TMD among college students [31]. Notably, being a morning person appears to be a protective factor against TMD, while being an evening person may increase the risk [31]. These observations align with our research and suggest that chronotypes could play a crucial role in the onset of TMD. Understanding this relationship offers valuable perspectives for the prevention and management of TMD in clinical settings. This connection hints that understanding a patient’s chronotype might aid in developing more tailored management strategies for TMD, offering a deeper insight into individualized care that considers natural sleep-wake preferences.

Insufficient sleep is linked to more severe pain and poorer self-reported health throughout life [32]. Patients with TMD frequently experience poor sleep quality and short sleep duration [33, 34]. Research has consistently demonstrated a significant association between sleep duration and TMD-pain, indicating that insufficient sleep can increase pain sensitivity and worsen clinical outcomes in TMD patients. Biologically, sleep deprivation disrupts slow-wave sleep, a phase critical for pain regulatory processes [35]. Reduced slow-wave activity impairs descending inhibitory pain pathways mediated by the prefrontal cortex and amygdala, while amplifying pro-nociceptive signaling through elevated substance P and glutamate levels in the trigeminal ganglion [35]. Additionally, short sleep duration elevates pro-inflammatory cytokines (*e.g.*, Interleukin-6 (IL-6), Tumor Necrosis Factor α (TNF-α)) that sensitize peripheral and central pain pathways, potentially exacerbating TMD-pain [36, 37]. For instance, Kim *et al*. [33] found that sleep duration is crucial for the long-term prognosis of TMD. Patients who sleep less than 6 hours per night experience more pain from eccentric jaw movements and higher levels of comorbidity. Moreover, these patients tend to respond poorly to traditional TMD treatments in terms of pain reduction and functional improvement [33]. Another study also suggested that insufficient sleep may be a core risk factor for functional limitation in patients with painful TMD [10]. Our findings align with existing literature, showing a negative correlation between genetically predicted sleep duration and the risk of TMD-pain. Specifically, longer sleep duration appears to be a protective factor that reduces the risk of TMD-pain. This correlation underscores the importance of adequate sleep in managing and potentially alleviating TMD symptoms. Given the complex and multifactorial nature of TMD, these results provide valuable insights into the potential benefits of improving sleep duration for the prevention and management of TMD-pain. This underscores the potential benefit of promoting better sleep habits as part of a holistic approach to TMD treatment, where improving sleep duration may lead to better pain management and overall patient well-being.

Sleep disorders, particularly insomnia, are prevalent among patients with TMD [38]. Numerous studies have suggested that insomnia-related sleep deprivation induces a proinflammatory state and enhances pain processing, leading to pain chronicity and increased morbidity [39, 40]. The bidirectional relationship between TMD-pain and insomnia may involve dysregulation of the hypothalamic-pituitary-adrenal (HPA) axis. Chronic pain activates the HPA axis, increasing cortisol secretion, which in turn disrupts sleep architecture by reducing Rapid eye movement (REM) sleep and fragmenting slow-wave sleep [41]. Furthermore, TMD-pain may amplify glutamatergic transmission in the thalamocortical circuits, perpetuating both hyperalgesia and sleep instability [42]. This may occur because insomnia and sleep disorders contribute to central sensitization, which is believed to play a significant role in the pathophysiology of TMD and other idiopathic chronic pain conditions [43]. For example, a recent study found that female TMD patients with objectively measured short sleep duration insomnia experienced more severe pain symptoms compared to those with normal sleep, potentially due to the upregulation of circulating IL-6 levels [44]. However, our MR results revealed no significant association between genetically predicted insomnia and sleep disorders and the risk of TMD-pain. Conversely, our findings indicated that genetically predicted TMD-pain increases the risk of insomnia and sleep disorders. These results provide new insights into the specific causal relationship between TMD-pain and insomnia and sleep disorders, highlighting the need for further research to clarify their complex association. These results highlight the need to address both TMD-pain and sleep disorders in clinical practice, as the interplay between the two conditions may exacerbate the overall patient experience. Recognizing this bidirectional relationship could inform comprehensive treatment strategies that address both pain management and sleep improvement.

For individuals with OSA, the upper airway intermittently collapses either completely or partially, leading to a suspension or reduction in airflow despite continued respiratory efforts [45]. This condition is only resolved when the central nervous system activates to restore airway patency. OSA is characterized by sleep fragmentation and nocturnal hypoxemia, which inevitably result in various complications and their sequelae [46]. The observed reverse causality—TMD-pain increasing OSA risk—may involve biomechanical and neuroinflammatory mechanisms. Chronic TMD-pain can induce compensatory forward head posture to reduce jaw loading, narrowing the upper airway and predisposing to collapse during sleep [45]. Additionally, TMD-related inflammation may upregulate cyclooxygenase-2 (COX-2) and prostaglandin E2 in the retrotemporal region, contributing to local tissue edema and airway resistance [47]. A substantial body of evidence indicates a close association between OSA and TMD. For instance, observational studies by Kang *et al*. [45] demonstrated an increased incidence of OSA in TMD patients compared to normal individuals, with TMD patients showing a heightened sensitivity to pain when they also have OSA. Additionally, a prospective cohort study by Sanders *et al*. [48] revealed that OSA occurrence is linked to a higher incidence of chronic TMD, with OSA symptoms often preceding the initial onset of TMD. However, our study did not find that genetically predicted OSA increases the risk of TMD or TMD-pain. Instead, it suggests that TMD-pain may increase the risk of developing OSA. This finding provides new insights into the complex relationship between OSA, TMD and TMD-pain, indicating the need for further research to clarify their causal connections. This unexpected finding warrants further investigation into the complex relationship between OSA and TMD-pain, and it suggests that clinicians managing TMD-pain may need to consider the potential for coexisting sleep apnea, offering a more integrative care approach to improve patient outcomes.

This study aimed to elucidate the causal relationship between seven sleep characteristics and TMD/TMD-pain. However, this study has several limitations. First, due to the limited number of SNPs meeting the stringent selection threshold (*p* < 5 × 10^−8^) in GWAS data related to OSA, sleep disorders, TMD and TMD-pain, we relaxed the significance threshold (*p* < 5 × 10^−6^) in these traits’ MR analyses, which may affect the robustness of causal inference. Second, although we utilized data from multiple large GWAS datasets, subgroup analyses were not feasible due to the lack of detailed demographic and clinical information. Furthermore, while our sensitivity analyses generally indicated minimal influence of horizontal pleiotropy, we observed a relatively low MR-PRESSO Global Test *p*-value of 0.063 in the association between TMD-pain and sleep disorders. Although this result does not reach the conventional significance threshold (*p* < 0.05), its proximity to the cutoff suggests a potential presence of horizontal pleiotropy. Therefore, caution is warranted in interpreting the causal inference between TMD-pain and sleep disorders. Future studies should incorporate larger GWAS sample sizes and improved pleiotropy correction methods to further validate these findings. Additionally, while we conducted multiple sensitivity analyses, including MR-Egger, weighted median and simple weighted model estimates, to reduce potential bias, the influence of horizontal pleiotropy and unmeasured confounding factors cannot be entirely ruled out. Fourth, a key limitation of this study is that the sleep-related data used in our MR analysis were derived from self-reported measures in large-scale biobanks (UK Biobank and FinnGen) rather than from standardized clinical diagnostic tools or validated sleep assessment questionnaires. While these datasets have been widely utilized in sleep-related GWAS studies, self-reported sleep traits may be subject to recall bias and inconsistencies in subjective reporting, which could impact the robustness of the instrumental variables and introduce some degree of measurement variability in causal estimates. However, given the large sample sizes and the genetic approach used in this study, MR remains a powerful tool for inferring causal relationships in epidemiology. Fifth, there is a potential for racial bias, as the study population primarily consisted of individuals of European origin, which may limit the generalizability of our findings to other populations. Therefore, further studies are necessary to validate our results and explore their potential integration into clinical practice for the development of diagnostic and therapeutic strategies.

## 5. Conclusions

This study provides evidence supporting a bidirectional association between sleep behaviors and TMD, though the causal interpretation should be made cautiously due to the reliance on self-reported sleep data. Our findings suggest that a genetic predisposition to being a morning person reduces the risk of TMD. Additionally, there is an inverse causal relationship between genetically predicted sleep duration and TMD-pain. Conversely, TMD-pain increases the risk of insomnia, OSA and sleep disorders. These findings underscore the importance of addressing sleep issues in TMD management. Further research is needed to validate these results across diverse populations and explore the underlying mechanisms. Integrating sleep-focused strategies into TMD management could improve patient outcomes and quality of life.

## Supplementary Material

Supplementary material associated with this article can be found, in the online version, at https://files.jofph.com/files/article/1933038893044514816/attachment/Supplyment%20material.docx.


