# Supplementary material for: Causal effects of sleep behaviors on temporomandibular disorders and pain: a bidirectional mendelian randomization study
Source: J Oral Facial Pain Headache. 2025 Jun 12;39(2):101–11. doi: 10.22514/jofph.2025.029 (PMC12531579; doi:10.22514/jofph.2025.029)
Supplement: Supplementary file 1 [file Supplementary-material.docx]

Supplementary material

Supplementary Table 1. Details of the number of genetic instruments and *F*-statistic for each exposure (SNPs with *p* < 5 ×10^−8^).

| Exposures | nSNPs | *F*-statistic (mean) | SNP | *F*-statistic |
| --- | --- | --- | --- | --- |
| Daytime napping | 88 | 47.50 | rs1001817 | 40.78 |
|  |  |  | rs10149986 | 48.78 |
|  |  |  | rs10152428 | 32.47 |
|  |  |  | rs10257273 | 39.69 |
|  |  |  | rs10811438 | 33.72 |
|  |  |  | rs10835420 | 40.27 |
|  |  |  | rs10840017 | 36.45 |
|  |  |  | rs10875606 | 31.82 |
|  |  |  | rs10875622 | 72.45 |
|  |  |  | rs11071755 | 33.55 |
|  |  |  | rs112520848 | 31.48 |
|  |  |  | rs11258652 | 52.95 |
|  |  |  | rs11615756 | 217.42 |
|  |  |  | rs11682175 | 32.71 |
|  |  |  | rs11860072 | 52.46 |
|  |  |  | rs12042846 | 32.14 |
|  |  |  | rs12140153 | 136.46 |
|  |  |  | rs12346996 | 34.03 |
|  |  |  | rs12451365 | 49.94 |
|  |  |  | rs12657723 | 39.71 |
|  |  |  | rs12992648 | 32.30 |
|  |  |  | rs13033444 | 52.06 |
|  |  |  | rs13150944 | 42.34 |
|  |  |  | rs13263535 | 31.91 |
|  |  |  | rs13284688 | 101.10 |
|  |  |  | rs140506252 | 30.89 |
|  |  |  | rs1546977 | 45.79 |
|  |  |  | rs1601440 | 45.31 |
|  |  |  | rs17158413 | 41.87 |
|  |  |  | rs17265513 | 36.11 |
|  |  |  | rs174541 | 61.22 |
|  |  |  | rs17502738 | 32.36 |
|  |  |  | rs1883048 | 41.41 |
|  |  |  | rs1931175 | 38.72 |
|  |  |  | rs2033103 | 35.37 |
|  |  |  | rs2099810 | 40.11 |
|  |  |  | rs2202323 | 36.38 |
|  |  |  | rs224111 | 40.85 |
|  |  |  | rs2250377 | 105.68 |
|  |  |  | rs2284015 | 30.93 |
|  |  |  | rs2370926 | 41.42 |
|  |  |  | rs2431108 | 101.24 |
|  |  |  | rs253666 | 32.26 |
|  |  |  | rs2653349 | 126.00 |
|  |  |  | rs2699869 | 31.01 |
|  |  |  | rs2769916 | 44.41 |
|  |  |  | rs2786547 | 45.99 |
|  |  |  | rs285815 | 38.10 |
|  |  |  | rs2943023 | 33.33 |
|  |  |  | rs295278 | 40.21 |
|  |  |  | rs34262487 | 37.98 |
|  |  |  | rs35011311 | 43.15 |
|  |  |  | rs350785 | 43.86 |
|  |  |  | rs351776 | 38.29 |
|  |  |  | rs35851551 | 30.51 |
|  |  |  | rs3799380 | 34.58 |
|  |  |  | rs3810484 | 31.34 |
|  |  |  | rs385199 | 208.09 |
|  |  |  | rs3935190 | 42.90 |
|  |  |  | rs4604518 | 32.05 |
|  |  |  | rs4653052 | 30.22 |
|  |  |  | rs467897 | 53.95 |
|  |  |  | rs4692709 | 34.92 |
|  |  |  | rs60222088 | 42.53 |
|  |  |  | rs60920123 | 38.75 |
|  |  |  | rs614987 | 77.84 |
|  |  |  | rs62189006 | 33.22 |
|  |  |  | rs6452787 | 40.34 |
|  |  |  | rs6919087 | 67.58 |
|  |  |  | rs73817091 | 29.85 |
|  |  |  | rs7422655 | 32.30 |
|  |  |  | rs7423968 | 38.81 |
|  |  |  | rs75022160 | 31.21 |
|  |  |  | rs76257331 | 32.51 |
|  |  |  | rs7697461 | 34.42 |
|  |  |  | rs77154532 | 36.66 |
|  |  |  | rs7814873 | 32.54 |
|  |  |  | rs785145 | 32.53 |
|  |  |  | rs7932966 | 37.35 |
|  |  |  | rs80163246 | 39.96 |
|  |  |  | rs908442 | 69.19 |
|  |  |  | rs910187 | 33.94 |
|  |  |  | rs9287862 | 32.43 |
|  |  |  | rs9309116 | 33.61 |
|  |  |  | rs9389556 | 37.03 |
|  |  |  | rs9460110 | 34.21 |
|  |  |  | rs9475168 | 35.26 |
|  |  |  | rs962247 | 42.26 |
|  |  |  | rs971415 | 35.73 |
|  |  |  | rs9939355 | 35.20 |
| Daytime sleepiness | 36 | 42.19 | rs11123962 | 60.48 |
|  |  |  | rs11942333 | 30.10 |
|  |  |  | rs12140153 | 85.18 |
|  |  |  | rs12153518 | 42.21 |
|  |  |  | rs13010456 | 54.23 |
|  |  |  | rs13097760 | 30.86 |
|  |  |  | rs13135092 | 30.28 |
|  |  |  | rs147114641 | 41.04 |
|  |  |  | rs1566362 | 34.97 |
|  |  |  | rs17131124 | 37.02 |
|  |  |  | rs17356118 | 39.32 |
|  |  |  | rs1846644 | 117.43 |
|  |  |  | rs2048522 | 30.67 |
|  |  |  | rs2787120 | 31.93 |
|  |  |  | rs285793 | 42.68 |
|  |  |  | rs3122170 | 60.00 |
|  |  |  | rs4665972 | 39.16 |
|  |  |  | rs4765939 | 36.01 |
|  |  |  | rs501701 | 34.16 |
|  |  |  | rs55818482 | 59.70 |
|  |  |  | rs55960940 | 31.84 |
|  |  |  | rs57746981 | 40.07 |
|  |  |  | rs62055936 | 43.65 |
|  |  |  | rs62519825 | 33.89 |
|  |  |  | rs6741951 | 35.68 |
|  |  |  | rs6897863 | 38.01 |
|  |  |  | rs6923811 | 37.57 |
|  |  |  | rs7476897 | 45.46 |
|  |  |  | rs7598712 | 30.92 |
|  |  |  | rs7607363 | 33.66 |
|  |  |  | rs7837226 | 30.91 |
|  |  |  | rs8015449 | 35.81 |
|  |  |  | rs825127 | 32.78 |
|  |  |  | rs843372 | 44.34 |
|  |  |  | rs886114 | 31.64 |
|  |  |  | rs960986 | 44.95 |
|  |  |  | rs9712275 | 32.51 |
| Insomnia | 11 | 24.25 | rs112162210 | 21.08 |
|  |  |  | rs112436795 | 21.98 |
|  |  |  | rs113265030 | 21.99 |
|  |  |  | rs115490521 | 25.74 |
|  |  |  | rs118104214 | 22.50 |
|  |  |  | rs138538976 | 21.36 |
|  |  |  | rs1402901 | 28.61 |
|  |  |  | rs2952019 | 24.48 |
|  |  |  | rs4858789 | 23.87 |
|  |  |  | rs7623125 | 27.31 |
|  |  |  | rs8015310 | 27.81 |
| Morning person | 111 | 45.34 | rs10067113 | 36.31 |
|  |  |  | rs10123584 | 29.75 |
|  |  |  | rs10149448 | 34.80 |
|  |  |  | rs10196909 | 50.73 |
|  |  |  | rs1027742 | 35.65 |
|  |  |  | rs10495976 | 36.57 |
|  |  |  | rs10501087 | 38.05 |
|  |  |  | rs10520176 | 54.70 |
|  |  |  | rs10818834 | 39.89 |
|  |  |  | rs10976942 | 36.50 |
|  |  |  | rs11032362 | 73.69 |
|  |  |  | rs11152350 | 38.39 |
|  |  |  | rs11174781 | 47.77 |
|  |  |  | rs11229543 | 40.87 |
|  |  |  | rs1144566 | 168.51 |
|  |  |  | rs114870822 | 29.02 |
|  |  |  | rs115774037 | 31.46 |
|  |  |  | rs11580135 | 32.57 |
|  |  |  | rs11587758 | 56.81 |
|  |  |  | rs11588913 | 30.21 |
|  |  |  | rs11645898 | 34.85 |
|  |  |  | rs11670534 | 29.19 |
|  |  |  | rs11679484 | 40.81 |
|  |  |  | rs11712056 | 49.70 |
|  |  |  | rs11786306 | 37.88 |
|  |  |  | rs11841335 | 29.94 |
|  |  |  | rs12140153 | 37.89 |
|  |  |  | rs12498561 | 29.92 |
|  |  |  | rs12669911 | 39.91 |
|  |  |  | rs12682033 | 43.44 |
|  |  |  | rs12927162 | 83.52 |
|  |  |  | rs12969848 | 63.86 |
|  |  |  | rs13065394 | 35.94 |
|  |  |  | rs13255030 | 32.43 |
|  |  |  | rs13269289 | 32.32 |
|  |  |  | rs138964083 | 37.97 |
|  |  |  | rs139911 | 67.82 |
|  |  |  | rs1421085 | 82.06 |
|  |  |  | rs1470764 | 38.09 |
|  |  |  | rs1494185 | 32.45 |
|  |  |  | rs149611468 | 32.26 |
|  |  |  | rs1524472 | 31.11 |
|  |  |  | rs17374439 | 96.26 |
|  |  |  | rs17575798 | 47.56 |
|  |  |  | rs17682747 | 32.65 |
|  |  |  | rs1947198 | 33.34 |
|  |  |  | rs202157 | 64.71 |
|  |  |  | rs2072727 | 37.42 |
|  |  |  | rs2102506 | 38.48 |
|  |  |  | rs2239626 | 46.80 |
|  |  |  | rs231398 | 33.16 |
|  |  |  | rs2467109 | 30.44 |
|  |  |  | rs2518022 | 59.29 |
|  |  |  | rs2653343 | 124.67 |
|  |  |  | rs28380327 | 38.71 |
|  |  |  | rs2842638 | 44.25 |
|  |  |  | rs28458909 | 97.88 |
|  |  |  | rs28634184 | 32.94 |
|  |  |  | rs2893787 | 31.19 |
|  |  |  | rs2910032 | 55.78 |
|  |  |  | rs2949923 | 34.37 |
|  |  |  | rs308521 | 36.51 |
|  |  |  | rs34581681 | 31.14 |
|  |  |  | rs34619169 | 31.17 |
|  |  |  | rs34627176 | 29.38 |
|  |  |  | rs34875688 | 35.97 |
|  |  |  | rs35653190 | 31.51 |
|  |  |  | rs3760185 | 49.48 |
|  |  |  | rs3767240 | 45.62 |
|  |  |  | rs3850174 | 37.57 |
|  |  |  | rs3877930 | 34.20 |
|  |  |  | rs4239386 | 51.40 |
|  |  |  | rs4321976 | 36.53 |
|  |  |  | rs4339281 | 33.85 |
|  |  |  | rs4729854 | 92.31 |
|  |  |  | rs4752593 | 32.15 |
|  |  |  | rs4822107 | 37.68 |
|  |  |  | rs4936291 | 33.89 |
|  |  |  | rs512647 | 29.11 |
|  |  |  | rs520954 | 77.61 |
|  |  |  | rs60194061 | 40.77 |
|  |  |  | rs60616179 | 31.86 |
|  |  |  | rs62046253 | 39.99 |
|  |  |  | rs62082401 | 57.05 |
|  |  |  | rs62553781 | 45.33 |
|  |  |  | rs6537834 | 29.74 |
|  |  |  | rs6599694 | 31.26 |
|  |  |  | rs6656331 | 36.14 |
|  |  |  | rs6744983 | 29.74 |
|  |  |  | rs6967481 | 43.72 |
|  |  |  | rs7001604 | 46.84 |
|  |  |  | rs72632979 | 33.30 |
|  |  |  | rs72720396 | 65.14 |
|  |  |  | rs72829936 | 33.25 |
|  |  |  | rs7302062 | 49.12 |
|  |  |  | rs7304278 | 41.82 |
|  |  |  | rs73606718 | 33.66 |
|  |  |  | rs75120545 | 49.19 |
|  |  |  | rs7547493 | 98.40 |
|  |  |  | rs75650221 | 30.35 |
|  |  |  | rs7602425 | 39.37 |
|  |  |  | rs769066 | 31.84 |
|  |  |  | rs7691121 | 40.82 |
|  |  |  | rs77008212 | 106.90 |
|  |  |  | rs7701529 | 31.80 |
|  |  |  | rs7735794 | 30.24 |
|  |  |  | rs778147 | 40.41 |
|  |  |  | rs786406 | 44.21 |
|  |  |  | rs7959983 | 48.38 |
|  |  |  | rs9369915 | 57.24 |
|  |  |  | rs9573971 | 75.63 |
|  |  |  | rs957501 | 30.38 |
|  |  |  | rs9597241 | 30.98 |
|  |  |  | rs9636202 | 30.08 |
|  |  |  | rs9962650 | 33.34 |
|  |  |  | rs9964420 | 68.06 |
| OSA | 38 | 32.87 | rs10094779 | 29.60 |
|  |  |  | rs10423928 | 33.39 |
|  |  |  | rs10507084 | 42.20 |
|  |  |  | rs10799768 | 25.96 |
|  |  |  | rs10928510 | 29.54 |
|  |  |  | rs10986730 | 37.03 |
|  |  |  | rs11075985 | 113.00 |
|  |  |  | rs113955098 | 39.44 |
|  |  |  | rs114106239 | 32.51 |
|  |  |  | rs11981973 | 38.79 |
|  |  |  | rs1228509 | 33.70 |
|  |  |  | rs12636366 | 29.56 |
|  |  |  | rs12924412 | 25.74 |
|  |  |  | rs13114985 | 31.48 |
|  |  |  | rs13333522 | 30.12 |
|  |  |  | rs139879148 | 28.59 |
|  |  |  | rs140896965 | 37.13 |
|  |  |  | rs1436341 | 29.11 |
|  |  |  | rs1808593 | 25.60 |
|  |  |  | rs1885767 | 25.86 |
|  |  |  | rs1959185 | 28.03 |
|  |  |  | rs2016950 | 30.08 |
|  |  |  | rs2170710 | 28.11 |
|  |  |  | rs2370982 | 33.28 |
|  |  |  | rs2530482 | 26.49 |
|  |  |  | rs4450466 | 25.62 |
|  |  |  | rs4809902 | 36.51 |
|  |  |  | rs5758238 | 27.68 |
|  |  |  | rs5769152 | 26.20 |
|  |  |  | rs59333125 | 32.30 |
|  |  |  | rs60700772 | 31.31 |
|  |  |  | rs61873510 | 30.53 |
|  |  |  | rs6484367 | 37.08 |
|  |  |  | rs679880 | 31.04 |
|  |  |  | rs72732695 | 27.28 |
|  |  |  | rs72981098 | 25.27 |
|  |  |  | rs76229479 | 35.43 |
|  |  |  | rs78189434 | 25.52 |
|  |  |  | rs935977 | 25.88 |
| Sleep disorder | 15 | 37.07 | rs10095135 | 31.82 |
|  |  |  | rs10270616 | 29.73 |
|  |  |  | rs10507084 | 38.59 |
|  |  |  | rs114106239 | 31.24 |
|  |  |  | rs12636366 | 33.43 |
|  |  |  | rs1959185 | 36.48 |
|  |  |  | rs1964272 | 33.07 |
|  |  |  | rs2064558 | 30.83 |
|  |  |  | rs2529273 | 31.65 |
|  |  |  | rs45551238 | 38.64 |
|  |  |  | rs6484367 | 33.24 |
|  |  |  | rs76229479 | 31.27 |
|  |  |  | rs8020365 | 29.88 |
|  |  |  | rs925966 | 31.36 |
|  |  |  | rs9940278 | 94.79 |
| Sleep duration | 60 | 40.39 | rs10173260 | 30.79 |
|  |  |  | rs10483350 | 36.69 |
|  |  |  | rs10761674 | 29.62 |
|  |  |  | rs1079727 | 34.74 |
|  |  |  | rs10973207 | 42.78 |
|  |  |  | rs11039544 | 35.79 |
|  |  |  | rs112230981 | 36.37 |
|  |  |  | rs113113059 | 34.77 |
|  |  |  | rs11567976 | 31.39 |
|  |  |  | rs11621908 | 33.50 |
|  |  |  | rs11643715 | 30.97 |
|  |  |  | rs11885663 | 38.37 |
|  |  |  | rs12246842 | 34.69 |
|  |  |  | rs12567114 | 34.08 |
|  |  |  | rs12607679 | 60.32 |
|  |  |  | rs12791153 | 31.18 |
|  |  |  | rs13088093 | 45.88 |
|  |  |  | rs13109404 | 50.10 |
|  |  |  | rs147114641 | 34.93 |
|  |  |  | rs151014368 | 32.57 |
|  |  |  | rs1517572 | 40.73 |
|  |  |  | rs1553132 | 31.50 |
|  |  |  | rs17427571 | 32.22 |
|  |  |  | rs174560 | 31.03 |
|  |  |  | rs17732997 | 31.95 |
|  |  |  | rs1776776 | 34.26 |
|  |  |  | rs1939455 | 32.89 |
|  |  |  | rs205024 | 35.30 |
|  |  |  | rs2072727 | 33.58 |
|  |  |  | rs2079070 | 46.75 |
|  |  |  | rs2192528 | 34.71 |
|  |  |  | rs2231265 | 30.70 |
|  |  |  | rs269054 | 35.39 |
|  |  |  | rs2696429 | 38.90 |
|  |  |  | rs2717076 | 61.85 |
|  |  |  | rs3027234 | 31.35 |
|  |  |  | rs3095508 | 44.38 |
|  |  |  | rs34354917 | 30.21 |
|  |  |  | rs35531607 | 31.92 |
|  |  |  | rs35662245 | 37.22 |
|  |  |  | rs365663 | 41.22 |
|  |  |  | rs374153 | 32.21 |
|  |  |  | rs4592416 | 41.83 |
|  |  |  | rs465700 | 37.56 |
|  |  |  | rs4767550 | 38.33 |
|  |  |  | rs4841498 | 38.04 |
|  |  |  | rs55658675 | 30.77 |
|  |  |  | rs56372231 | 49.85 |
|  |  |  | rs61985058 | 33.16 |
|  |  |  | rs62120041 | 32.57 |
|  |  |  | rs6575005 | 34.71 |
|  |  |  | rs7198661 | 34.62 |
|  |  |  | rs73219758 | 43.20 |
|  |  |  | rs7556815 | 220.86 |
|  |  |  | rs7644809 | 32.21 |
|  |  |  | rs7806045 | 31.73 |
|  |  |  | rs7915425 | 40.66 |
|  |  |  | rs8038326 | 39.26 |
|  |  |  | rs915416 | 59.60 |
|  |  |  | rs9345234 | 32.03 |
|  |  |  | rs9382445 | 38.79 |
|  |  |  | rs9937053 | 54.67 |
| TMD | 20 | 22.44 | rs10882591 | 27.66 |
|  |  |  | rs111843207 | 21.02 |
|  |  |  | rs114568702 | 23.55 |
|  |  |  | rs12466258 | 21.24 |
|  |  |  | rs138516336 | 22.09 |
|  |  |  | rs141765089 | 21.15 |
|  |  |  | rs16995253 | 20.91 |
|  |  |  | rs2058908 | 23.03 |
|  |  |  | rs2062550 | 23.02 |
|  |  |  | rs2360795 | 21.33 |
|  |  |  | rs56323002 | 21.44 |
|  |  |  | rs6752506 | 23.72 |
|  |  |  | rs67939396 | 26.24 |
|  |  |  | rs72935077 | 21.64 |
|  |  |  | rs7515572 | 24.11 |
|  |  |  | rs76537824 | 21.99 |
|  |  |  | rs76646321 | 20.86 |
|  |  |  | rs77640278 | 21.09 |
|  |  |  | rs78882783 | 21.19 |
|  |  |  | rs903554 | 21.52 |
| TMD-pain | 36 | 21.27 | rs117069883 | 19.66 |
|  |  |  | rs117115183 | 19.79 |
|  |  |  | rs11717864 | 22.45 |
|  |  |  | rs11772232 | 20.24 |
|  |  |  | rs12406887 | 22.00 |
|  |  |  | rs12901888 | 19.96 |
|  |  |  | rs139690482 | 20.13 |
|  |  |  | rs145527519 | 20.01 |
|  |  |  | rs146875287 | 23.00 |
|  |  |  | rs17079019 | 20.01 |
|  |  |  | rs1830821 | 24.22 |
|  |  |  | rs186586219 | 21.14 |
|  |  |  | rs2101358 | 27.74 |
|  |  |  | rs2206722 | 21.10 |
|  |  |  | rs2649888 | 20.03 |
|  |  |  | rs2797386 | 22.83 |
|  |  |  | rs2966867 | 23.34 |
|  |  |  | rs34641111 | 21.36 |
|  |  |  | rs4739605 | 19.62 |
|  |  |  | rs4807510 | 21.59 |
|  |  |  | rs59957178 | 20.87 |
|  |  |  | rs6092776 | 20.25 |
|  |  |  | rs61772601 | 21.05 |
|  |  |  | rs62221604 | 20.53 |
|  |  |  | rs62534851 | 22.34 |
|  |  |  | rs7009937 | 20.81 |
|  |  |  | rs72694955 | 25.39 |
|  |  |  | rs72806751 | 21.98 |
|  |  |  | rs75813347 | 20.93 |
|  |  |  | rs76976204 | 20.89 |
|  |  |  | rs77237649 | 19.70 |
|  |  |  | rs78079857 | 20.37 |
|  |  |  | rs78322646 | 20.10 |
|  |  |  | rs78882783 | 20.13 |
|  |  |  | rs8017299 | 20.56 |
|  |  |  | rs9969570 | 19.62 |

SNPs: Single Nucleotide Polymorphisms; OSA: Obstructive Sleep Apnea; TMD: Temporomandibular Disorder; TMD-pain: TMD related pain.

Supplementary Table 2. Bidirectional MR estimates of sleep behaviors and TMD/TMD-pain (SNPs with *p* < 5 ×10^−8^).

| Exposure | Outcome | Method | nSNPs | *p*val | beta | 95% CI | FDR_*q*val |
| --- | --- | --- | --- | --- | --- | --- | --- |
| Daytime napping | TMD | MR-Egger | 88 | 0.583 | 0.524 | −1.341, 2.389 | 0.978 |
|  |  | Weighted median | 88 | 0.794 | 0.097 | −0.631, 0.825 | 0.926 |
|  |  | Inverse variance weighted | 88 | 0.729 | −0.091 | −0.603, 0.422 | 0.877 |
|  |  | Simple mode | 88 | 0.876 | −0.144 | −1.948, 1.659 | 0.943 |
|  |  | Weighted mode | 88 | 0.597 | 0.407 | −1.097, 1.912 | 0.914 |
|  |  | BWMR | 88 | 0.539 | −0.169 | −0.707, 0.370 | 0.839 |
| Daytime sleepiness | TMD | MR-Egger | 36 | 0.757 | 0.730 | −3.853, 5.313 | 0.978 |
|  |  | Weighted median | 36 | 0.714 | 0.262 | −1.137, 1.661 | 0.908 |
|  |  | Inverse variance weighted | 36 | 0.980 | −0.013 | −1.052,1.025 | 0.980 |
|  |  | Simple mode | 36 | 0.658 | 0.580 | −1.961, 3.121 | 0.943 |
|  |  | Weighted mode | 36 | 0.535 | 0.711 | −1.512, 2.935 | 0.914 |
|  |  | BWMR | 36 | 0.931 | 0.044 | −0.951, 1.039 | 0.965 |
| Insomnia | TMD | MR-Egger | 11 | 0.406 | −0.172 | −0.559, 0.215 | 0.978 |
|  |  | Weighted median | 11 | 0.641 | 0.038 | −0.121, 0.197 | 0.897 |
|  |  | Inverse variance weighted | 11 | 0.783 | 0.018 | −0.111, 0.147 | 0.877 |
|  |  | Simple mode | 11 | 0.730 | 0.051 | −0.231, 0.333 | 0.943 |
|  |  | Weighted mode | 11 | 0.425 | 0.094 | −0.127, 0.315 | 0.914 |
|  |  | BWMR | 11 | 0.979 | 0.002 | −0.132, 0.135 | 0.979 |
| Morning person | TMD | MR-Egger | 111 | 0.507 | −0.146 | −0.576, 0.284 | 0.978 |
|  |  | Weighted median | 111 | 0.018 | −0.221 | −0.403, −0.038 | 0.035 |
|  |  | Inverse variance weighted | 111 | 0.014 | −0.173 | −0.311, −0.034 | 0.031 |
|  |  | Simple mode | 111 | 0.318 | −0.258 | −0.763, 0.246 | 0.743 |
|  |  | Weighted mode | 111 | 0.260 | −0.302 | −0.824, 0.221 | 0.614 |
|  |  | BWMR | 111 | 0.031 | −0.157 | −0.299, −0.015 | 0.047 |
| OSA | TMD | MR-Egger | 38 | 0.301 | −0.279 | −0.801, 0.242 | 0.978 |
|  |  | Weighted median | 38 | 0.190 | 0.143 | −0.071, 0.356 | 0.252 |
|  |  | Inverse variance weighted | 38 | 0.125 | 0.121 | −0.033, 0.275 | 0.289 |
|  |  | Simple mode | 38 | 0.209 | 0.328 | −0.175, 0.830 | 0.343 |
|  |  | Weighted mode | 38 | 0.236 | 0.358 | −0.225, 0.941 | 0.374 |
|  |  | BWMR | 38 | 0.093 | 0.133 | −0.022, 0.287 | 0.168 |
| Sleep disorder | TMD | MR-Egger | 15 | 0.394 | −0.445 | −1.434, 0.544 | 0.978 |
|  |  | Weighted median | 15 | 0.197 | 0.225 | −0.117, 0.568 | 0.552 |
|  |  | Inverse variance weighted | 15 | 0.520 | 0.096 | −0.196, 0.387 | 0.857 |
|  |  | Simple mode | 15 | 0.371 | 0.328 | −0.368, 1.023 | 0.943 |
|  |  | Weighted mode | 15 | 0.478 | 0.321 | −0.543, 1.185 | 0.914 |
|  |  | BWMR | 15 | 0.447 | 0.116 | −0.182, 0.413 | 0.736 |
| Sleep duration | TMD | MR-Egger | 60 | 0.657 | 0.339 | −1.151, 1.829 | 0.978 |
|  |  | Weighted median | 60 | 0.839 | −0.054 | −0.579, 0.470 | 0.939 |
|  |  | Inverse variance weighted | 60 | 0.636 | −0.090 | −0.464, 0.283 | 0.877 |
|  |  | Simple mode | 60 | 0.774 | −0.174 | −1.356, 1.009 | 0.943 |
|  |  | Weighted mode | 60 | 0.873 | −0.070 | −0.920, 0.780 | 0.989 |
|  |  | BWMR | 60 | 0.636 | −0.093 | −0.480, 0.293 | 0.876 |
| Daytime napping | TMD-pain | MR-Egger | 88 | 0.846 | 0.131 | −1.186, 1.447 | 0.978 |
|  |  | Weighted median | 88 | 0.978 | −0.008 | −0.573, 0.557 | 0.978 |
|  |  | Inverse variance weighted | 88 | 0.473 | 0.133 | −0.230, 0.496 | 0.837 |
|  |  | Simple mode | 88 | 0.823 | −0.159 | −1.551, 1.233 | 0.943 |
|  |  | Weighted mode | 88 | 0.951 | −0.038 | −1.233, 1.157 | 0.989 |
|  |  | BWMR | 88 | 0.640 | 0.089 | −0.284, 0.463 | 0.876 |
| Daytime sleepiness | TMD-pain | MR-Egger | 36 | 0.623 | 0.935 | −2.754, 4.624 | 0.978 |
|  |  | Weighted median | 36 | 0.914 | −0.059 | −1.128, 1.011 | 0.978 |
|  |  | Inverse variance weighted | 36 | 0.690 | 0.170 | −0.667, 1.007 | 0.877 |
|  |  | Simple mode | 36 | 0.868 | −0.166 | −2.104, 1.771 | 0.943 |
|  |  | Weighted mode | 36 | 0.984 | 0.018 | −1.746, 1.782 | 0.989 |
|  |  | BWMR | 36 | 0.703 | 0.156 | −0.648, 0.960 | 0.876 |
| Insomnia | TMD-pain | MR-Egger | 11 | 0.772 | 0.037 | −0.203, 0.277 | 0.978 |
|  |  | Weighted median | 11 | 0.609 | 0.029 | −0.083, 0.141 | 0.897 |
|  |  | Inverse variance weighted | 11 | 0.663 | 0.018 | −0.061, 0.097 | 0.877 |
|  |  | Simple mode | 11 | 0.739 | −0.032 | −0.213, 0.150 | 0.943 |
|  |  | Weighted mode | 11 | 0.904 | 0.011 | −0.165, 0.187 | 0.989 |
|  |  | BWMR | 11 | 0.710 | 0.017 | −0.074, 0.109 | 0.876 |
| Morning person | TMD-pain | MR-Egger | 111 | 0.846 | −0.029 | −0.321, 0.263 | 0.978 |
|  |  | Weighted median | 111 | 0.110 | −0.104 | −0.231, 0.023 | 0.752 |
|  |  | Inverse variance weighted | 111 | 0.077 | −0.085 | −0.179, 0.009 | 0.158 |
|  |  | Simple mode | 111 | 0.432 | −0.175 | −0.609, 0.259 | 0.943 |
|  |  | Weighted mode | 111 | 0.393 | −0.182 | −0.597, 0.234 | 0.914 |
|  |  | BWMR | 111 | 0.064 | −0.090 | −0.185, 0.005 | 0.255 |
| OSA | TMD-pain | MR-Egger | 38 | 0.824 | −0.041 | −0.394, 0.313 | 0.978 |
|  |  | Weighted median | 38 | 0.556 | 0.045 | −0.106, 0.196 | 0.895 |
|  |  | Inverse variance weighted | 38 | 0.478 | 0.037 | −0.065, 0.140 | 0.837 |
|  |  | Simple mode | 38 | 0.956 | 0.010 | −0.342, 0.361 | 0.956 |
|  |  | Weighted mode | 38 | 0.963 | 0.010 | −0.410, 0.430 | 0.989 |
|  |  | BWMR | 38 | 0.420 | 0.043 | −0.062, 0.148 | 0.735 |
| Sleep disorder | TMD-pain | MR-Egger | 15 | 0.932 | 0.028 | −0.608, 0.665 | 0.978 |
|  |  | Weighted median | 15 | 0.421 | 0.099 | −0.143, 0.341 | 0.752 |
|  |  | Inverse variance weighted | 15 | 0.363 | 0.083 | −0.096, 0.262 | 0.766 |
|  |  | Simple mode | 15 | 0.311 | 0.262 | −0.227, 0.751 | 0.943 |
|  |  | Weighted mode | 15 | 0.404 | 0.245 | −0.313, 0.804 | 0.914 |
|  |  | BWMR | 15 | 0.317 | 0.097 | −0.093, 0.288 | 0.682 |
| Sleep duration | TMD-pain | MR-Egger | 60 | 0.435 | −0.492 | −1.721, 0.736 | 0.978 |
|  |  | Weighted median | 60 | 0.321 | −0.202 | −0.600, 0.197 | 0.752 |
|  |  | Inverse variance weighted | 60 | 0.015 | −0.382 | −0.689, −0.075 | 0.035 |
|  |  | Simple mode | 60 | 0.689 | −0.199 | −1.166, 0.769 | 0.943 |
|  |  | Weighted mode | 60 | 0.927 | −0.032 | −0.711, 0.647 | 0.989 |
|  |  | BWMR | 60 | 0.013 | −0.389 | −0.696, −0.083 | 0.028 |
| TMD | Daytime napping | MR-Egger | 20 | 0.528 | 0.003 | −0.007, 0.013 | 0.978 |
|  |  | Weighted median | 20 | 0.791 | −0.001 | −0.008, 0.006 | 0.926 |
|  |  | Inverse variance weighted | 20 | 0.125 | 0.005 | −0.001, 0.010 | 0.389 |
|  |  | Simple mode | 20 | 0.381 | −0.006 | −0.019, 0.007 | 0.943 |
|  |  | Weighted mode | 20 | 0.520 | −0.003 | −0.011, 0.006 | 0.914 |
|  |  | BWMR | 20 | 0.050 | 0.004 | 0.002, 0.009 | 0.133 |
| TMD | Daytime sleepiness | MR-Egger | 20 | 0.062 | 0.007 | 0.000, 0.014 | 0.278 |
|  |  | Weighted median | 20 | 0.363 | 0.003 | −0.003, 0.008 | 0.752 |
|  |  | Inverse variance weighted | 20 | 0.244 | 0.003 | −0.002, 0.007 | 0.569 |
|  |  | Simple mode | 20 | 0.820 | 0.001 | −0.008, 0.010 | 0.943 |
|  |  | Weighted mode | 20 | 0.517 | 0.002 | −0.004, 0.008 | 0.914 |
|  |  | BWMR | 20 | 0.273 | 0.002 | −0.001, 0.005 | 0.637 |
| TMD | Insomnia | MR-Egger | 20 | 0.382 | −0.084 | −0.267, 0.099 | 0.978 |
|  |  | Weighted median | 20 | 0.962 | −0.004 | −0.150, 0.143 | 0.978 |
|  |  | Inverse variance weighted | 20 | 0.600 | 0.029 | −0.079, 0.137 | 0.877 |
|  |  | Simple mode | 20 | 0.943 | −0.010 | −0.265, 0.246 | 0.956 |
|  |  | Weighted mode | 20 | 0.989 | −0.002 | −0.246, 0.243 | 0.989 |
|  |  | BWMR | 20 | 0.856 | 0.011 | −0.110, 0.132 | 0.922 |
| TMD | Morning person | MR-Egger | 20 | 0.912 | 0.003 | −0.051, 0.057 | 0.978 |
|  |  | Weighted median | 20 | 0.284 | −0.013 | −0.036, 0.011 | 0.752 |
|  |  | Inverse variance weighted | 20 | 0.383 | −0.014 | −0.046, 0.018 | 0.766 |
|  |  | Simple mode | 20 | 0.773 | −0.006 | −0.043, 0.032 | 0.943 |
|  |  | Weighted mode | 20 | 0.426 | −0.011 | −0.038, 0.016 | 0.914 |
|  |  | BWMR | 20 | 0.789 | −0.003 | −0.024, 0.019 | 0.920 |
| TMD | OSA | MR-Egger | 20 | 0.365 | 0.060 | −0.067, 0.187 | 0.978 |
|  |  | Weighted median | 20 | 0.397 | 0.025 | −0.033, 0.083 | 0.752 |
|  |  | Inverse variance weighted | 20 | 0.874 | 0.006 | −0.067, 0.079 | 0.927 |
|  |  | Simple mode | 20 | 0.643 | −0.024 | −0.123, 0.075 | 0.943 |
|  |  | Weighted mode | 20 | 0.807 | −0.012 | −0.108, 0.084 | 0.989 |
|  |  | BWMR | 20 | 0.401 | 0.024 | −0.032, 0.080 | 0.735 |
| TMD | Sleep disorder | MR-Egger | 20 | 0.390 | 0.052 | −0.063, 0.167 | 0.978 |
|  |  | Weighted median | 20 | 0.418 | 0.022 | −0.031, 0.075 | 0.752 |
|  |  | Inverse variance weighted | 20 | 0.729 | 0.012 | −0.054, 0.077 | 0.877 |
|  |  | Simple mode | 20 | 0.599 | −0.031 | −0.142, 0.081 | 0.943 |
|  |  | Weighted mode | 20 | 0.609 | −0.028 | −0.136, 0.079 | 0.914 |
|  |  | BWMR | 20 | 0.357 | 0.024 | −0.027, 0.075 | 0.713 |
| TMD | Sleep duration | MR-Egger | 20 | 0.415 | −0.008 | −0.026, 0.011 | 0.978 |
|  |  | Weighted median | 20 | 0.356 | 0.005 | −0.006, 0.017 | 0.752 |
|  |  | Inverse variance weighted | 20 | 0.197 | 0.008 | −0.004, 0.020 | 0.502 |
|  |  | Simple mode | 20 | 0.591 | 0.004 | −0.012, 0.021 | 0.943 |
|  |  | Weighted mode | 20 | 0.418 | 0.005 | −0.007, 0.018 | 0.914 |
|  |  | BWMR | 20 | 0.094 | 0.007 | −0.001, 0.016 | 0.268 |
| TMD-pain | Daytime napping | MR-Egger | 36 | 0.992 | 0.000 | −0.010, 0.010 | 0.992 |
|  |  | Weighted median | 36 | 0.430 | 0.003 | −0.005, 0.011 | 0.752 |
|  |  | Inverse variance weighted | 36 | 0.144 | 0.005 | −0.002, 0.012 | 0.404 |
|  |  | Simple mode | 36 | 0.109 | 0.012 | −0.002, 0.026 | 0.943 |
|  |  | Weighted mode | 36 | 0.620 | 0.002 | −0.006, 0.011 | 0.914 |
|  |  | BWMR | 36 | 0.196 | 0.005 | −0.002, 0.012 | 0.499 |
| TMD-pain | Daytime sleepiness | MR-Egger | 36 | 0.943 | 0.000 | −0.007, 0.007 | 0.978 |
|  |  | Weighted median | 36 | 0.268 | 0.003 | −0.003, 0.009 | 0.752 |
|  |  | Inverse variance weighted | 36 | 0.106 | 0.004 | −0.001, 0.008 | 0.389 |
|  |  | Simple mode | 36 | 0.650 | 0.002 | −0.008, 0.013 | 0.943 |
|  |  | Weighted mode | 36 | 0.426 | 0.003 | −0.004, 0.010 | 0.914 |
|  |  | BWMR | 36 | 0.096 | 0.004 | −0.001, 0.009 | 0.268 |
| TMD-pain | Insomnia | MR-Egger | 36 | 0.241 | 0.133 | −0.086, 0.352 | 0.978 |
|  |  | Weighted median | 36 | 0.073 | 0.138 | −0.013, 0.288 | 0.681 |
|  |  | Inverse variance weighted | 36 | 0.013 | 0.140 | 0.030, 0.250 | 0.027 |
|  |  | Simple mode | 36 | 0.602 | 0.089 | −0.243, 0.422 | 0.943 |
|  |  | Weighted mode | 36 | 0.558 | 0.105 | −0.243, 0.454 | 0.914 |
|  |  | BWMR | 36 | 0.020 | 0.142 | 0.022, 0.262 | 0.036 |
| TMD-pain | Morning person | MR-Egger | 36 | 0.420 | −0.013 | −0.045, 0.019 | 0.978 |
|  |  | Weighted median | 36 | 0.575 | −0.007 | −0.033, 0.018 | 0.895 |
|  |  | Inverse variance weighted | 36 | 0.894 | 0.001 | −0.019, 0.022 | 0.927 |
|  |  | Simple mode | 36 | 0.385 | 0.024 | −0.029, 0.077 | 0.943 |
|  |  | Weighted mode | 36 | 0.200 | −0.021 | −0.053, 0.011 | 0.914 |
|  |  | BWMR | 36 | 0.719 | 0.004 | −0.019, 0.027 | 0.876 |
| TMD-pain | OSA | MR-Egger | 36 | 0.677 | 0.021 | −0.077, 0.118 | 0.978 |
|  |  | Weighted median | 36 | 0.259 | 0.035 | −0.026, 0.097 | 0.752 |
|  |  | Inverse variance weighted | 36 | 0.013 | 0.063 | 0.013, 0.114 | 0.029 |
|  |  | Simple mode | 36 | 0.532 | 0.046 | −0.097, 0.189 | 0.943 |
|  |  | Weighted mode | 36 | 0.568 | 0.036 | −0.086, 0.158 | 0.914 |
|  |  | BWMR | 36 | 0.021 | 0.061 | 0.009, 0.112 | 0.038 |
| TMD-pain | Sleep disorder | MR-Egger | 36 | 0.472 | 0.031 | −0.052, 0.114 | 0.978 |
|  |  | Weighted median | 36 | 0.042 | 0.057 | 0.002, 0.112 | 0.088 |
|  |  | Inverse variance weighted | 36 | 0.001 | 0.070 | 0.027, 0.113 | 0.010 |
|  |  | Simple mode | 36 | 0.013 | 0.184 | 0.046, 0.322 | 0.035 |
|  |  | Weighted mode | 36 | 0.761 | 0.020 | −0.107, 0.146 | 0.989 |
|  |  | BWMR | 36 | 0.002 | 0.070 | 0.026, 0.115 | 0.014 |
| TMD-pain | Sleep duration | MR-Egger | 36 | 0.543 | −0.007 | −0.028, 0.015 | 0.978 |
|  |  | Weighted median | 36 | 0.685 | −0.003 | −0.017, 0.011 | 0.908 |
|  |  | Inverse variance weighted | 36 | 0.762 | 0.002 | −0.012, 0.016 | 0.877 |
|  |  | Simple mode | 36 | 0.526 | −0.008 | −0.033, 0.016 | 0.943 |
|  |  | Weighted mode | 36 | 0.539 | −0.005 | −0.020, 0.010 | 0.914 |
|  |  | BWMR | 36 | 0.825 | −0.002 | −0.015, 0.012 | 0.922 |

TMD: Temporomandibular Disorder; MR: Mendelian Randomization; BWMR: Bayesian Weighted Mendelian Randomization; OSA: Obstructive Sleep Apnea; TMD-pain: TMD related pain; SNPs: Single Nucleotide Polymorphisms; CI: Confidence Interval; FDR: false discovery rate; *p*val: *p* value; *q*val: *q* value.

Supplementary Table 3. Heterogeneity and horizontal pleiotropy tests of MR estimates of sleep behaviors and TMD/TMD-pain.

| Exposure | Outcome | *Q* *p*val  (IVW) | *Q* *p*val  (MR-Egger) | Intercept | Intercept *p*val | MR-PRESSO Global Test *p*val |
| --- | --- | --- | --- | --- | --- | --- |
| Daytime napping | TMD | 0.267 | 0.280 | −0.006 | 0.503 | 0.278 |
| Daytime sleepiness | TMD | 0.244 | 0.278 | −0.006 | 0.746 | 0.248 |
| Insomnia | TMD | 0.173 | 0.162 | 0.071 | 0.334 | 0.165 |
| Morning person | TMD | 0.153 | 0.163 | −0.001 | 0.898 | 0.144 |
| OSA | TMD | 0.199 | 0.150 | 0.024 | 0.125 | 0.148 |
| Sleep disorder | TMD | 0.068 | 0.078 | 0.031 | 0.283 | 0.080 |
| Sleep duration | TMD | 0.174 | 0.189 | −0.007 | 0.562 | 0.187 |
| Daytime napping | TMD-pain | 0.497 | 0.528 | 0.002 | 0.997 | 0.525 |
| Daytime sleepiness | TMD-pain | 0.070 | 0.083 | −0.006 | 0.679 | 0.082 |
| Insomnia | TMD-pain | 0.411 | 0.502 | −0.007 | 0.873 | 0.509 |
| Morning person | TMD-pain | 0.111 | 0.122 | −0.002 | 0.693 | 0.127 |
| OSA | TMD-pain | 0.492 | 0.529 | 0.005 | 0.656 | 0.502 |
| Sleep disorder | TMD-pain | 0.200 | 0.255 | 0.003 | 0.863 | 0.207 |
| Sleep duration | TMD-pain | 0.092 | 0.105 | 0.002 | 0.857 | 0.152 |
| TMD | Daytime napping | 0.018 | 0.025 | 0.004 | 0.762 | 0.075 |
| TMD | Daytime sleepiness | 0.231 | 0.156 | −0.001 | 0.131 | 0.192 |
| TMD | Insomnia | 0.363 | 0.293 | 0.025 | 0.159 | 0.291 |
| TMD | Morning person | 0.308 | 0.242 | −0.005 | 0.443 | 0.126 |
| TMD | OSA | 0.210 | 0.175 | −0.012 | 0.320 | 0.063 |
| TMD | Sleep disorder | 0.098 | 0.138 | −0.009 | 0.415 | 0.060 |
| TMD | Sleep duration | 0.146 | 0.185 | 0.004 | 0.052 | 0.195 |
| TMD-pain | Daytime napping | 0.121 | 0.102 | 0.001 | 0.222 | 0.402 |
| TMD-pain | Daytime sleepiness | 0.115 | 0.097 | 0.001 | 0.222 | 0.113 |
| TMD-pain | Insomnia | 0.374 | 0.420 | 0.013 | 0.945 | 0.435 |
| TMD-pain | Morning person | 0.065 | 0.067 | 0.002 | 0.247 | 0.061 |
| TMD-pain | OSA | 0.270 | 0.240 | 0.006 | 0.325 | 0.103 |
| TMD-pain | sleep disorder | 0.137 | 0.134 | 0.005 | 0.291 | 0.063 |
| TMD-pain | Sleep duration | 0.466 | 0.414 | 0.002 | 0.298 | 0.143 |

OSA: Obstructive Sleep Apnea; TMD: Temporomandibular Disorder; TMD-pain: TMD related pain; IVW: Inverse Variance Weighting; MR: Mendelian Randomization; BWMR: Bayesian Weighted Mendelian Randomization. *p*val: *p* value.


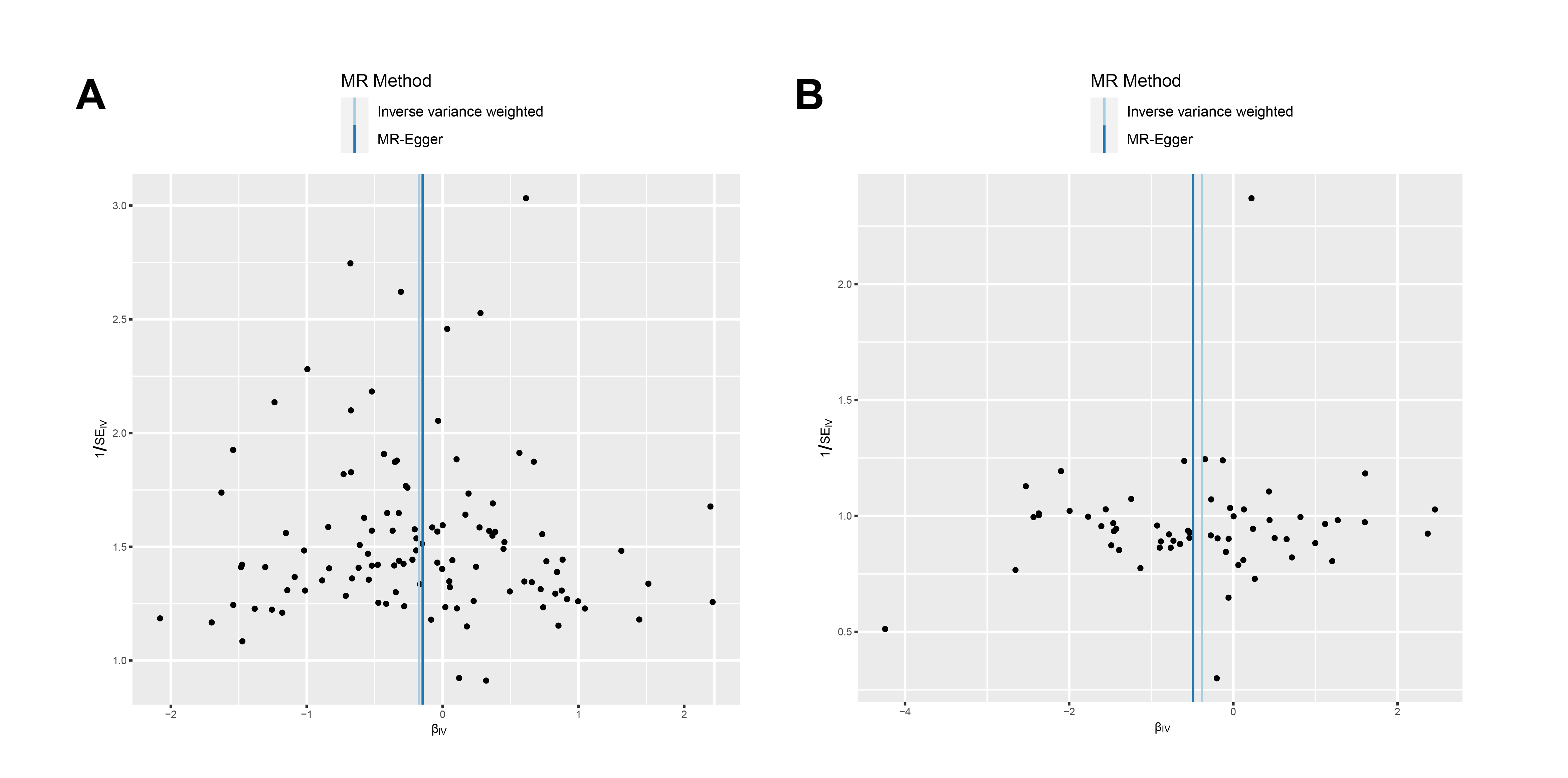


**Supplementary Fig. 1. The funnel plot of the impact of sleep behaviors on TMD/TMD-pain.** (A) The funnel plot of the impact of morning person on TMD. (B) The funnel plot of the impact of sleep duration on TMD-pain. MR: Mendelian Randomization; SE_IV_: Standard Error of the Instrumental Variable.


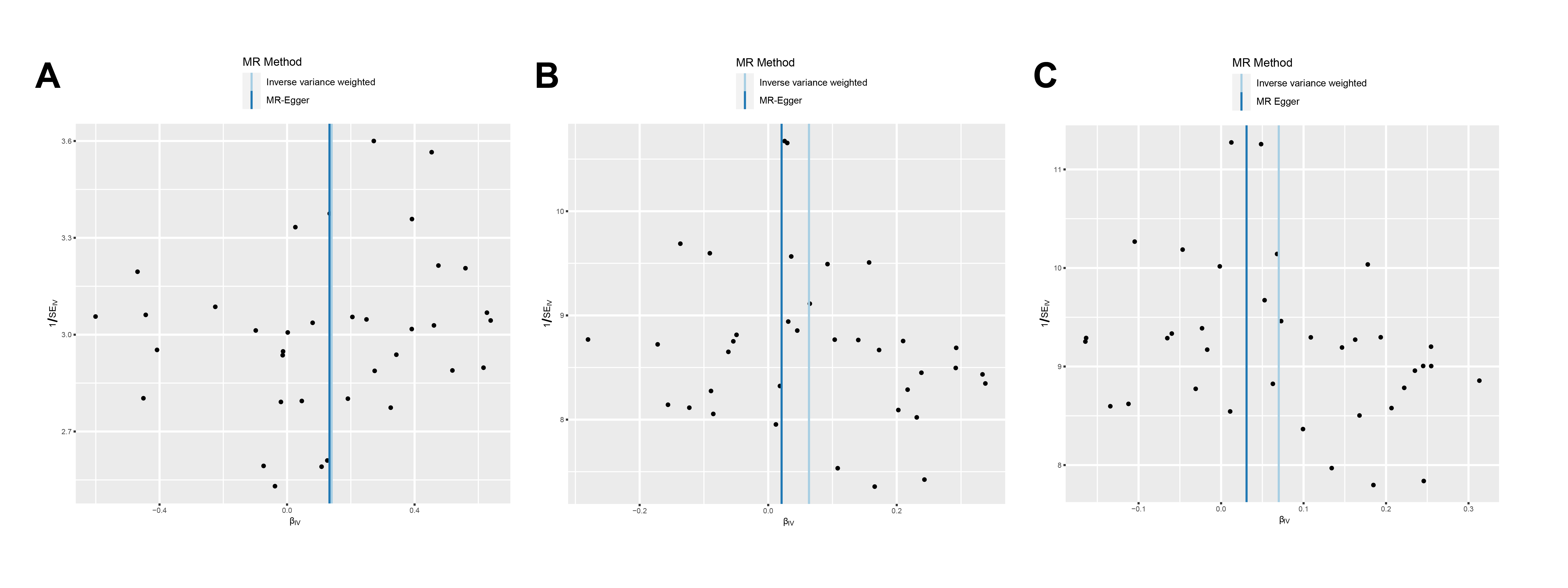


**Supplementary Fig. 2. The funnel plot of the impact of TMD/TMD-pain on sleep behaviors.** (A) The funnel plot of the impact of insomnia on TMD-pain. (B) The funnel plot of the impact of OSA on TMD-pain. (C) The funnel plot of the impact of sleep disorder on TMD-pain. MR: Mendelian Randomization; SE_IV_: Standard Error of the Instrumental Variable.


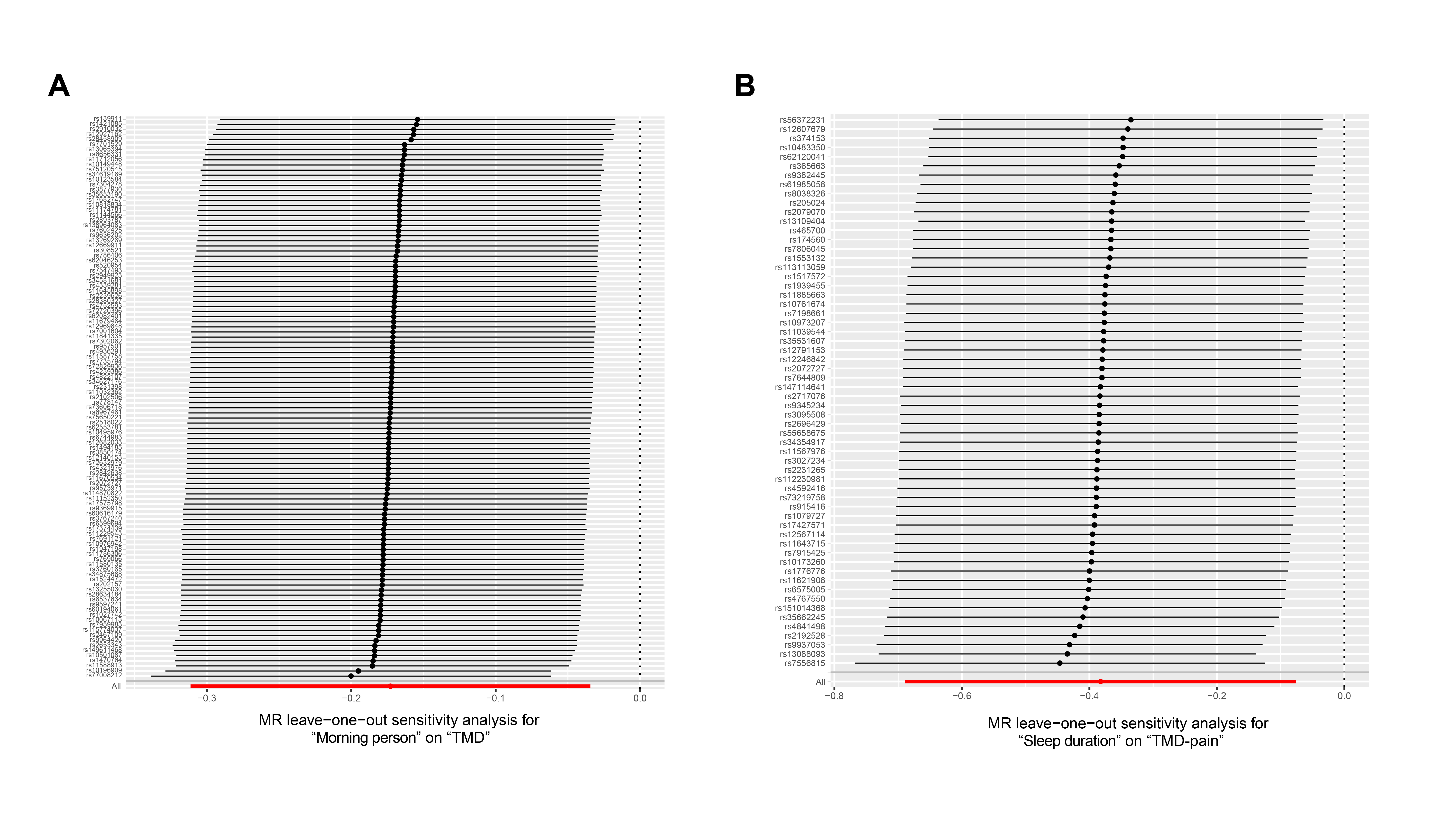


**Supplementary Fig. 3.** **The leave one out plot of the impact of sleep behaviors on TMD/TMD-pain.** (A) The leave one out plot of the impact of morning person on TMD. (B) The leave one out plot of the sleep duration on TMD-pain. MR: Mendelian Randomization; TMD: Temporomandibular Disorder; TMD-pain: TMD related pain.


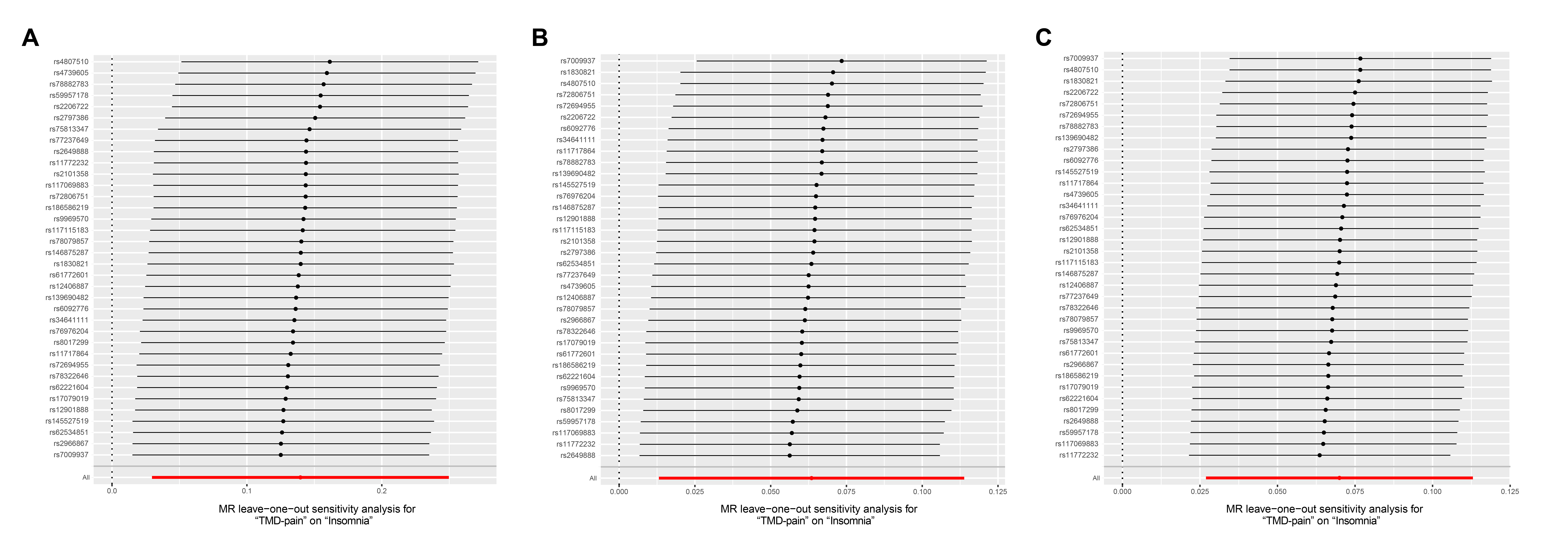


**Supplementary Fig. 4. The leave one out plot of the impact of TMD/TMD-pain on sleep behaviors.** (A) The leave one out plot of the impact of insomnia on TMD-pain. (B) The leave one out plot of the impact of OSA on TMD-pain. (C) The leave one out plot of the impact of sleep disorder on TMD-pain. MR: Mendelian Randomization; TMD-pain: TMD related pain.
